# Fatal pneumonia caused by *Penicillium digitatum*: a case report

**DOI:** 10.1186/1471-2466-13-16

**Published:** 2013-03-23

**Authors:** Chiyako Oshikata, Naomi Tsurikisawa, Akemi Saito, Maiko Watanabe, Yoichi Kamata, Maki Tanaka, Takahiro Tsuburai, Hiroyuki Mitomi, Kosuke Takatori, Hiroshi Yasueda, Kazuo Akiyama

**Affiliations:** 1 Clinical Research Centre for Allergy and Rheumatology, National Hospital Organization, Sagamihara Hospital, 18-1 Sakuradai, Minami-ku, Sagamihara, Kanagawa, 252-0392, Japan; 2 Division of Microbiology, National Institute of Health Science, 1-18-1 Kamiyoga, Setagaya-ku, Tokyo, 158–8501, Japan; 3 Centre for Fungal Consultation, 5F 4-8-5, Chuo, Tsurumi-ku, Yokohama, Kanagawa, 230-0051, Japan

**Keywords:** *Penicillium digitatum*, *Penicillium* species, Infection, Immunocompromised host, Pulmonary emphysema, Pneumonia

## Abstract

**Background:**

*Penicillium* species are among the most common fungi present in the environment and are usually considered non-pathogenic to humans. However, in immunocompromised hosts they can be virulent pathogens and can cause death. *Penicillium digitatum* is a plant pathogen that commonly causes a postharvest fungal disease of citrus called green mould; it very rarely causes systemic mycosis in humans. Here, we report a case of fatal pneumonia due to *P. digitatum* infection, as confirmed by repeated examination of cultured sputum.

**Case presentation:**

A cavity was found in the left upper lung on routine chest X-ray in a 78-year-old undernourished male who had been diagnosed at age 66 with bronchial asthma and pulmonary emphysema. No increased sputum production was present. The presence of antigen-specific precipitating antibodies to *Aspergillus flavus* and *P. digitatum* was confirmed in the patient’s serum and also later pleural fluid by using Ouchterlony double immunodiffusion testing with *A. flavus* and *P. digitatum* antigens. The patient was treated over a period of months with itraconazole, micafungin, voriconazole, amphotericin B, and antibacterials. However, the cavity enlarged, the pleural effusion increased, and the patient began producing purulent sputum. He died from progressive renal failure. From sputum culture only one fungus was isolated repeatedly on potato-dextrose agar in large quantities. This fungus was confirmed to be *P. digitatum* by molecular identification. Partial sequences of the beta-tubulin gene were determined by using the primers Bt2a and Bt2b for PCR amplification and sequencing and underwent a BLAST search at the National Centre for Biotechnology Information, these results confirmed that the isolated fungus was *P. digitatum*.

**Conclusion:**

To our knowledge, this is the first report of pulmonary infection with *P. digitatum*. Our patient had pulmonary emphysema and was elderly, and undernourished. These factors might have facilitated the infection. In his case, antimycotics were ineffective in treating the lung involvement. Although human infection with *P. digitatum* is considered rare, it appears that this organism can be very virulent and resistant to antimycotics.

## Background

*Penicillium* species are among the most common fungi in the environment and are usually considered non-pathogenic to humans [1]. However, in immunocompromised hosts they can be virulent pathogens that can cause death [2]. *Penicillium digitatum* is a plant pathogen that commonly causes a postharvest fungal disease of citrus called green mould [1]; it very rarely causes systemic mycosis in humans [3]. Here, we report a case of fatal pneumonia due to *P. digitatum* infection, as confirmed by repeated examination of cultured sputum.

## Case presentation

**Figure 1:**
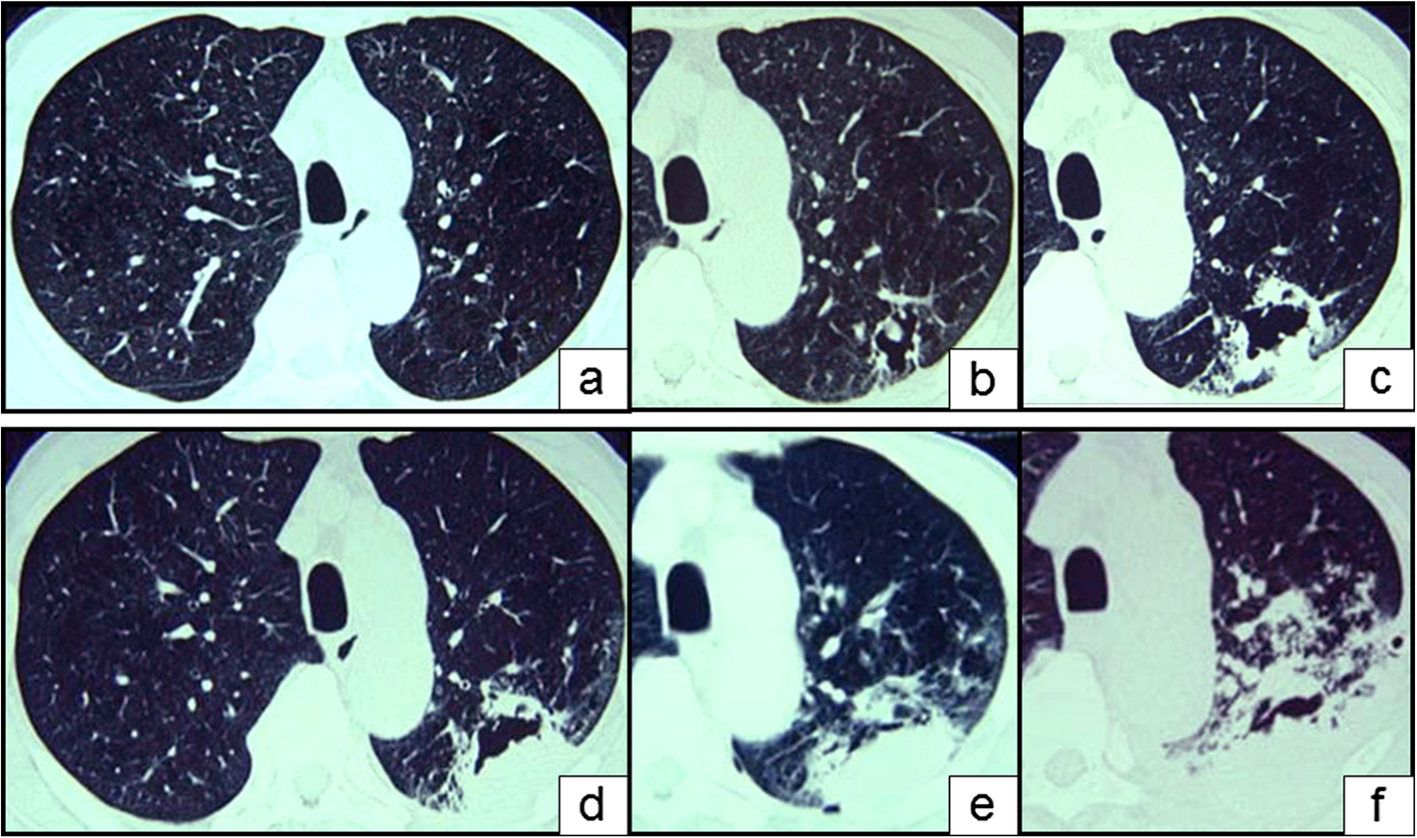
**Computed tomographic imaging of the chest at the level of the aortic arch in 2003, two years before dyspnea onset, showed a small cavity in the left upper lobe due to emphysematous change (a).** Computed tomography 2 years after initial presentation (April 2005) showed that the cavity was increasing in size and contained a fungus ball (**b**). The cavity then continued to increase in size, developing a thick wall and infiltration of the surrounding areas, with increased pulmonary effusion (**c**: July 2005; **d**: October 2005; **e**: December 2005; **f**: January 2006).

A 78-year-old male presented at our hospital in April 2005 with a history of bronchial asthma and pulmonary emphysema first diagnosed at age 66 years. He had been an office worker for 40 years and had never been involved in agriculture. He had therefore had no obvious opportunity for exposure to the citrus pathogen in his work environment or in and around his house. His asthma was of the non-atopic type and moderate, as defined by the Global Initiative for Asthma Guidelines 2002. The patient was an ex-smoker with a Brinkman Index of 1590. He was being treated with inhaled corticosteroids and theophylline. On first presentation in April 2005 to our hospital, he did not have asthma exacerbation or increased sputum production, but his dyspnoea on effort was graded 2 on the Hugh–Jones scale. In April 2005, when the patient was 78 years old, an abnormal shadow representing a cavity was found in the left upper lung on chest X ray at his yearly medical check-up. At the time the patient did not have increased sputum production, but chest computed tomography (CT) revealed a thin-walled cavity about 4 cm across and containing a fungus ball in the left upper lobe (S1 + 2); a CT scan taken 2 years previously had revealed only a small cavity indicative of emphysematous change (Figures 1a, 1b). There were no inflammatory changes in the peripheral blood (leukocyte count, 7950 cells/μL; C-reactive protein, 0.23 mg/dL; erythrocyte sedimentation rate, 10 mm/h; *Aspergillus* antigen, negative; β-D glucan, negative), but antigen-specific precipitating antibodies to *Aspergillus flavus* and *P. digitatum* were confirmed in the patient’s serum and pleural fluid by Ouchterlony double immunodiffusion testing [4]. No *A. flavus* or *P. digitatum* and no bacteria or tubercle bacilli were detected in cultures of sputum or bronchial lavage fluids. We diagnosed the patient with lung aspergilloma and treated with itraconazole (100 mg/day) for 3 months. However, the cavity became larger and thicker-walled (Figure 1c, July 2005), and the patient developed back pain. He was admitted to our hospital on 25 July 2005 and was treated for 3 months with an increased dose of itraconazole (200 mg/day) with added micafungin (300 mg/day). The patient’s vital capacity (VC) of 2.64 L, percentage VC of 85.7%, forced expiratory volume in 1 s (FEV1) of 1.09 L, and percentage FEV1 of 50.9% in August 2005 was lower than his VC of 3.01 L,%VC of 95.6%, FEV1 of 1.12 L,%FEV1 of 49.6% in 2003. The patient was unable to undergo further lung function tests because of his progressive respiratory failure.

**Figure 2:**
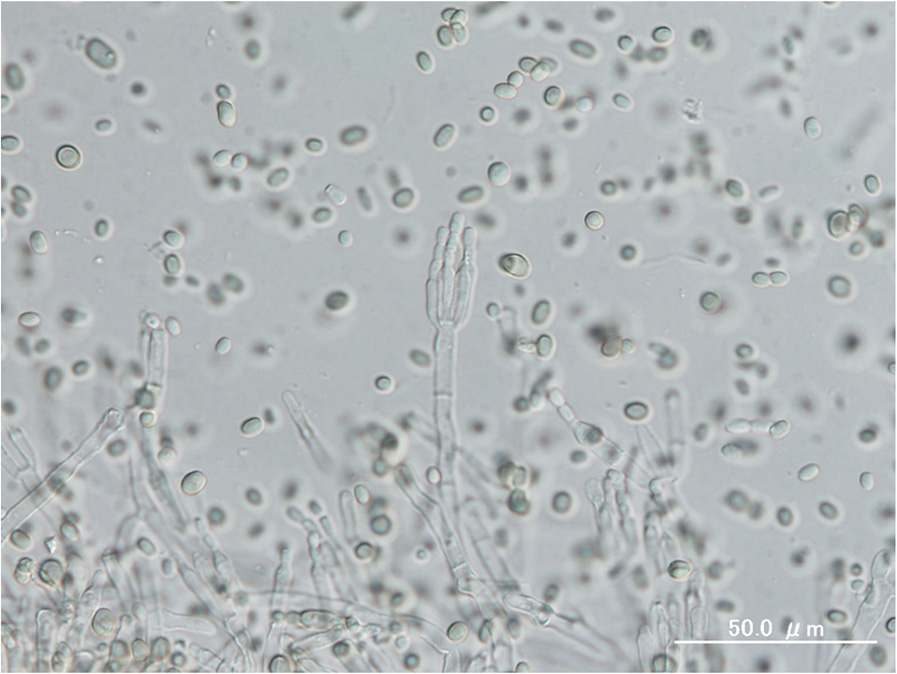
**The fruiting structures (penicilli) of *Penicillium *from this patient have a brush-like appearance; the spores of *Penicillium digitatum *are typically elliptical under the microscope.**

The cavity continued to enlarge further. Its fluid content increased, and consolidation appeared around it (Figure 1d, October 2005). The patient’s medication regimen was changed to voriconazole (400 mg/day), amphotericin B (10 mg/day), and fluconazole (400 mg/day), in addition to itraconazole (200 mg/day) and antibacterials. Treatment with this broad range of antimycotics and antibiotics did not slow the growth of the cavity: its fluid content continued to increase, and invasive consolidation and pleural effusion developed (Figure 1e, December 2005; 1f, January 2006). The pleural effusion increased, and the patient began producing purulent sputum. He died in February 2006 from progressive renal failure. Sputum samples yielded a single fungus, which was isolated repeatedly on potato-dextrose agar in large quantities. It was identified as *P. digitatum* and had the form of a spreading organism with a mealy, grey-green colour that turned olive green in culture. The abundance of the organism’s elliptical spores was greater in the patient’s sputum culture (Figure 2) than in cultured reference colonies [1]. This fungus was confirmed to be *P. digitatum* by molecular identification. Partial sequences of the β-tubulin gene determined by using the primers Bt2a and Bt2b [5] underwent BLAST analysis at the National Centre for Biotechnology Information.

**Figure 3:**
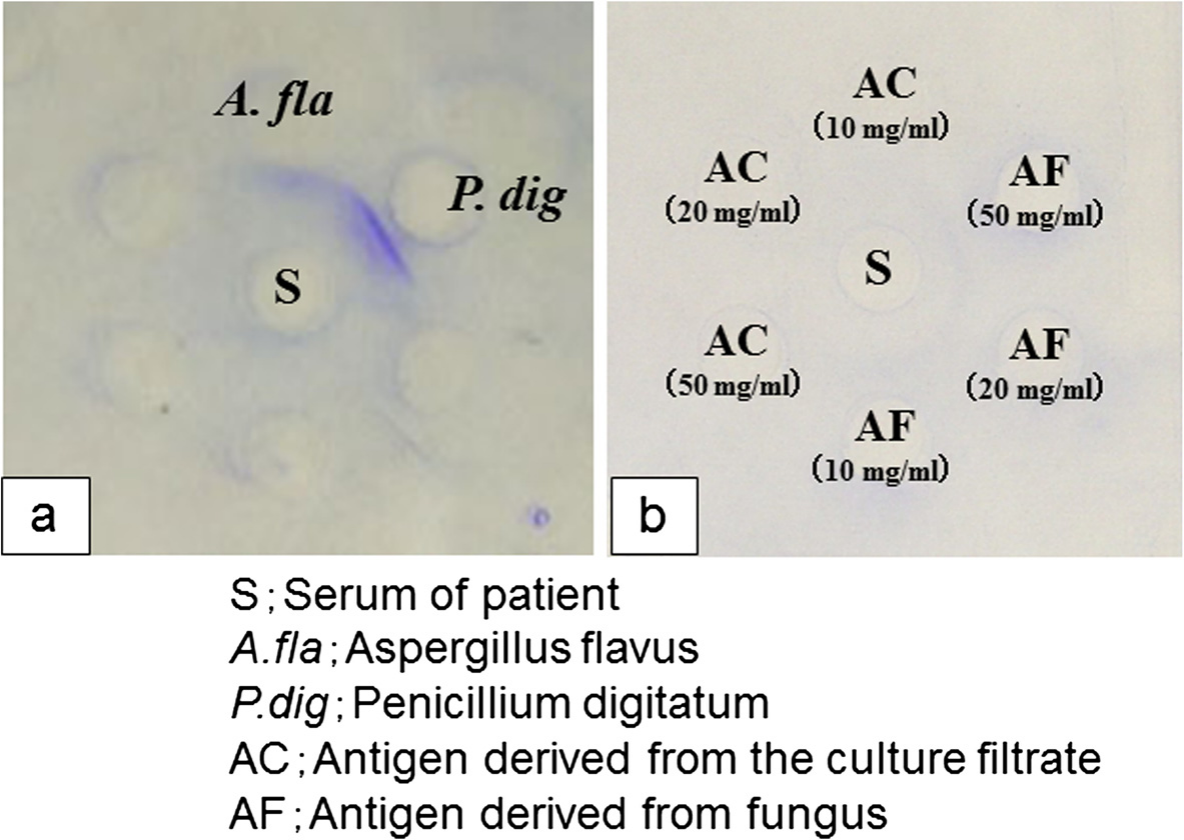
**Antigen-specific precipitating antibodies to *Aspergillus flavus *and *Penicillium digitatum *in the patient’s serum (a) were found by Ouchterlony double immunodiffusion testing with *A. flavus *and *P. digitatum *antigens.** We confirmed the presence of antigen-specific precipitating antibodies to *P. digitatum* by using antigen derived from the patient’s sputum culture fluid or extracted from the fungus in his sputum (**b**).

We found antigen-specific precipitating antibodies to *A. flavus* and *P. digitatum* in the patient’s serum (Figure 3a) at April 2005.and pleural effusion at November 2005 by using Ouchterlony double immunodiffusion testing [4] with *A. flavus* and *P. digitatum* antigens (HollisterStier, Spokane, WA, USA). We confirmed the presence of antigen-specific precipitating antibodies to *P digitatum* by using antigen derived from the patient’s sputum culture fluid or extracted directly from his sputum (Figure 3b).

To extract the antigen from the sputum culture, we added 1.5 mL of Glass Beads (Biospec Product, OK, USA) to the patient’s sputum and crushed the mixture with a Mini-Beadbeater (Biospec Product, OK, USA). It was then incubated with 0.125 mol of NH_4_CO_3_ overnight at 4°C and the antigen extracted after freeze-drying of the filtrate. We diagnosed invasive pulmonary penicilliosis due to *P. digitatum.*

## Discussion

*Penicillium digitatum* is the most devastating pathogen of rotten citrus fruit and is responsible for 90% of production losses during post-harvest handling [6]. *Penicillium digitatum* is widely distributed in soils throughout the world. People are commonly exposed to the spores of this airborne pathogen every day. Upon Ouchterlony double immunodiffusion testing, the sera of five of 770 patients with pulmonary lung disease were confirmed to have low titres of antigen-specific precipitating antibodies to *P. digitatum* against antigen (obtained from Greer Laboratories, Lenoir, NC). However, none of these patients had antigen-specific precipitating antibodies to *P. digitatum* in tests of antigen extracted from patient’ sputum culture fluid or sputum. In humans there is cross-reactivity to *Aspergillus* and *Penicillium*: most sera from patients with precipitins against *Penicillium* have precipitins against *Aspergillus*[7]. In April 2005, our patient had precipitins against both *P. digitatum* and *A. flavus*, but we did not find any *P. digitatum* or *A. flavus* in sputum cultures or in bronchoalveolar lavage fluids. This led to a delay in diagnosis.

*Penicillium* species can cause opportunistic infections [2]. Patients with *Penicillium* species infections have been treated successfully with itraconazole [8], amphotericin B [3,9], or fluconazole [3]. However, some patients with conditions caused by *Penicillium* species have died despite treatment with ketoconazole [2], amphotericin B [2], or itraconazole [10]. Pulmonary infections with fungi, including *Penicillium* species, are associated with much higher mortality rates in patients with nosocomial infections or infections complicating organ failure [11]. The minimal inhibitory concentrations of amphotericin B, itraconazole, posaconazole, ravuconazole, and voriconazole against *A. flavus* or *Aspergillus fumigatus* are lower than those against *Penicillium* spp. [12].

## Conclusions

To our knowledge, this is the first report of pulmonary infection with *P. digitatum*. Our patient had pulmonary emphysema and was elderly, and undernourished. He had no known history of exposure to citrus fruit pathogens, but he was likely to have been immunocompromised. These factors might have facilitated the infection. In his case, antimycotics were ineffective in treating the lung involvement. Although human infections with *P. digitatum* are considered rare, it appears that this organism can be very virulent and resistant to antimycotics.

## Consent

Written informed consent was obtained from the kin of the patient for publication of this Case Report and any accompanying images. A copy of the written consent is available for review by the Editor-in-Chief of this journal form.

## Competing interests

The authors declare that they have no competing interests.

## Authors’ contributions

CO examined the patient and contributed to manuscript preparation. NT examined the patient, took part in discussions about the patient, and was involved in manuscript preparation and editing. AS performed the tests for antigen-specific precipitating antibodies to *A. flavus* and *P. digitatum* in the patient’s serum. MW and YK confirmed the fungus as *P. digitatum* by molecular identification. KT and MT identified the fungus as *P. digitatum*, TT, HY, HM and KA contributed to discussions about the patient. All authors read and approved the final manuscript.

## Pre-publication history

The pre-publication history for this paper can be accessed here:

http://www.biomedcentral.com/1471-2466/13/16/prepub
